# Predicting the risk of portal vein thrombosis in patients with liver cirrhosis and hepatocellular carcinoma

**DOI:** 10.1016/j.heliyon.2020.e04677

**Published:** 2020-08-20

**Authors:** Waleed Mohamed Serag, Bedoor Shehap eldeen Mohammed, Magdy Mahmoud Mohamed, Basem Eysa Elsayed

**Affiliations:** a Department of Chemistry, Faculty of Science, Suez University, Suez, Egypt; b Chemical Administration, Suez, Egypt; c Department of Biochemistry, Faculty of Science, Ain Shams University, Cairo, Egypt; d National Hepatology and Tropical Medicine Research Institute, Cairo, Egypt

**Keywords:** Inflammation, Pathophysiology, Hematological system, Immunology, Laboratory medicine, Clinical research, Portal vein thrombosis, PhosphatidylSerine bearing microparticles (PS+MPs) levels, Plasma AnxA5 level, Plasma annexin A5 /PS+MP ratio, Portal flow velocity

## Abstract

The mechanisms of the hypercoagulable state in cirrhotics with and without hepatocellular carcinoma are incompetently comprehended. Objective: We aimed to explore the plasma Annexin A5/PS + MP ratio in these patients. Higher levels of Annexin A5 and PhosphatidylSerine bearing microparticles have been observed in cases of inflammation and increased coagulation but there are no studies which explore if there is an association between them and PVT in cirrhotics with and without HCC. So, our goal is to estimate their role in predicting PVT within HCV cirrhotics with and without HCC. 91 HCV cirrhotics with and without HCC and 20 healthy people (controls) were enlisted. Cirrhotics with and without HCC who developed PVT displayed higher levels of PS + MPs and lower Annexin A5/PS + MPs ratio (38.73 ± 1.92) and (0.00238 ± 0.00047) than cirrhotics who didn't develop PVT (22.19 ± 10.58) and (0.00451 ± 0.0023) (P < 0.001). Among the tested factors, lower Annexin A5/PS + MPs ratio show higher performance in predicting PVT in total cirrhotics, AUC, 0.919 followed by PS + MPs level, 0.876, Portal flow velocity, 0.842, Plasma Annexin A5 level, 0.509. In our hypothesis, As phosphatidylserine exposure increase due to increased level of circulating microparticles in cirrhotics with and without HCC, anenxin-A5 may be secreted by platelets and endothelial cells into the circulation as a physiological response to inactivate the elevated levels of PS bearing MPs produced in these patients but the increase in anenxin-A5 level isn't equivalent to the increase in PS bearing MPs levels. The equilibrium between plasma annexin A5 and PS bearing MPs levels is defected.

## 1 Introduction

Portal vein thrombosis is the obstruction of the portal vein or its branches by a blood thrombus [1]. Its prevalence is 1% in the commonalty [2] and will increase with the intensity of cirrhosis. So, It is about 1 percent in compensated liver cirrhosis, up to twenty-eight percent in decompensated liver cirrhosis, and may increase up to forty four percent when there is an association between liver cirrhosis and malignancies, particularly HCC [3]. Cirrhotics with HCC are strange as the Hemostasis disturbs towards a prothrombotic state due to the prescence of both conditions cancer and liver cirrhosis. To realize how Hepatocellular carcinoma can disturb the hemostasis, the status in which HCC progress must be considered, with this HCC nearly being a consequence of liver cirrhosis. The imbalanced hemostatic status of cirrhosis can be easily tend to thrombosis by the compound cases, including HCC [4]. Currently, no optimal modalities are found to monitor PVT. Studies have shown that we can prognosticate the progression of PVT in cirrhotsic from a hypercoagulable condition, decrease in the blood flow rate, endothelial cell injury, and cirrhosis complications, but the conclusions are incompatible [5]. Biomarkers of thrombosis risk might aid in choosing patients who can achieve the most benefit from anticoagulant medication and obviate exposing patients with low risk of thrombosis to the anticoagulant complications [6]. We hypothesized that disproportion between plasma annexin A5 and PhosphatidylSerine bearing microparticles (PS + MPs) levels could at least slightly clarify this hypercoagulability. Increased MP levels derived from different cells were detected in cirrhotics with and without HCC [7, 8]. Microparticles are extracellular vesicles extensively studied in hemostasis due to the capability to carry on their surface TF or negatively charged phosphatidylserine (PS) [9, 10]. Their excretion is the resullt of many inflammatory stimulants such as cancer-related inflammation and endotoxemia [11]. Annexin A5 (anxA5) is a distinctive member of the Annexin group known by its ability to bind to phosphatidylserine with great affinity in a ca^+2^ dependent manner [12]. AnxA5 has both extracellular and intracellular presence, particularly in platelets and endothelial cells [13]. The presence of PS on MPs is enough to enhance annexin binding of A5. Thus, inhibiting the procoagulant activity of PS bearing MPs [14]. High blood levels of annexin V reflect the elevated rate of cell death and the intensity of cell damage [15, 16]. Higher plasma levels of AnxA5 have also been detected in hypertension patients [17] and in other cases related to increased coagulation states and inflammation such as SCD [18] and SLE [19]. Our goal was to examine the presence of PhosphatidylSerine bearing microparticles (PS + MPs) and Annexin A5 in plasma of HCV cirrhotics with and without HCC and to explore if the lower plasma Annexin A5/PS + MP ratio in these patients could predict the development of PVT.

## 2 Materials and methods

**Figure 1:**
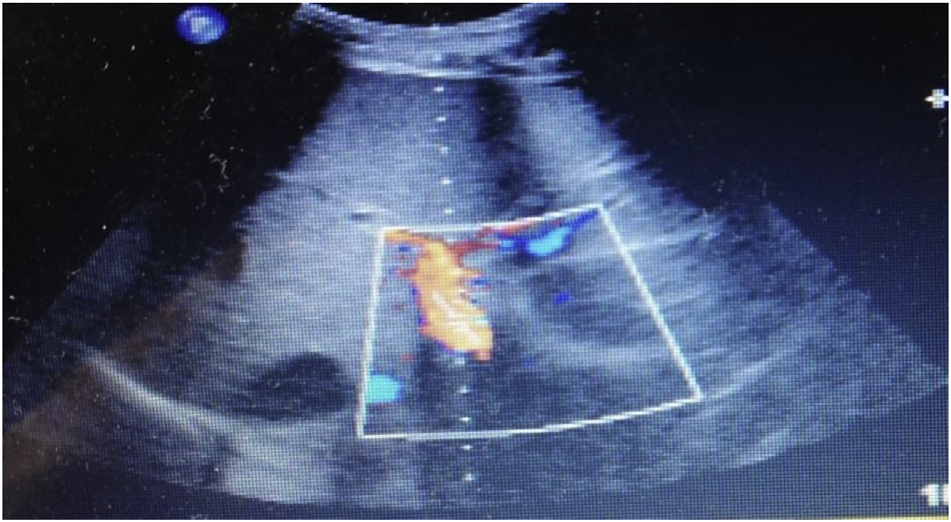
Image of Doppler US represents normal portal vein (no portal vein thrombosis).

**Figure 2:**
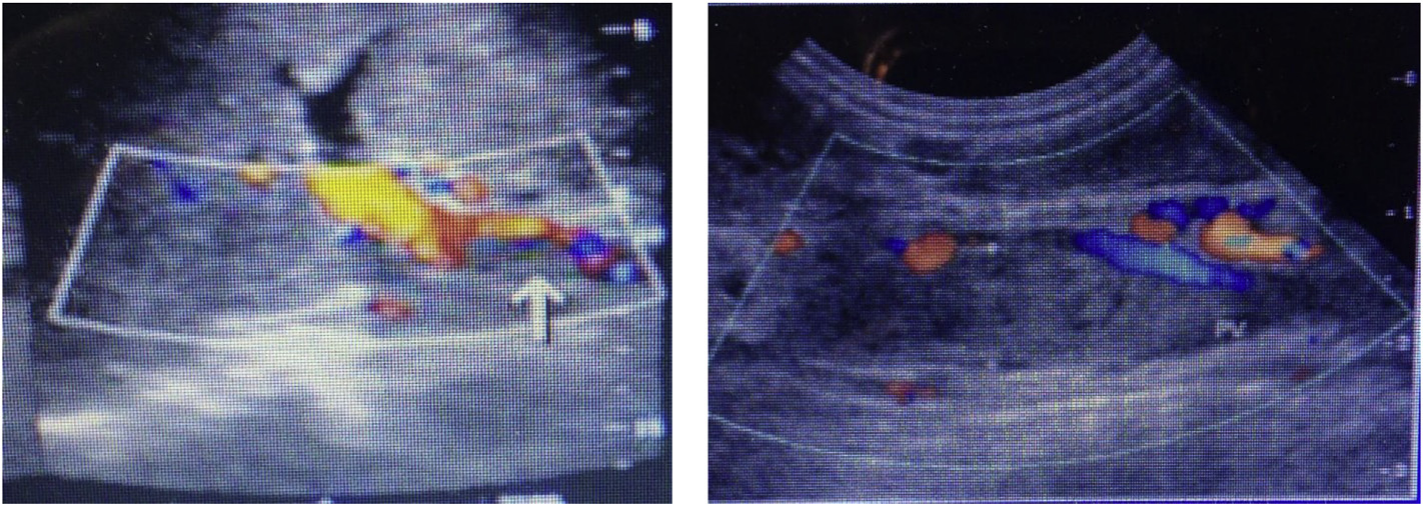
Images of Doppler US represent portal vein thrombosis.

This longitudinal study comprised 47 HCV cirrhotic patients without HCC (27 were males and 20 were females), 44 HCV cirrhotic patients with HCC (23 were males and 21 were females) and 20 individuals (10 were males and 10 were females) with no evidence of liver diseases admitted to the Egyptian National Hepatology & Tropical Medicine Research Institute (NHTMRI) within the period from March 2017 to August 2018. Formal consent was obtained from all studied individuals. The study was approved by the ethics committee of NHTMRI and registered under serial number 15–2016. The study was organized according to the Declaration of Helsinki for human subject research. Histological screening of liver biopsy, Doppler ultrasound, and unambiguous laboratory changes were carried out to all the patients for prope diagnosis. The intensity of liver disease was estimated with reference to the MELD score and the Pugh-Child score. The criteria of inclusion were clinical affirmation of hepatitis C-related cirrhosis with and without HCC by using imaging and laboratory analysis depending on the AASLD practice guidelines [20]. The exclusion criteria were Patients on antiplatelets, marivan and other thrombolytic agents, patients with established PVT at the beginning of the study, Patients with inherited coagulation abnormalities, Patients with renal dysfunction, Patients with splenectomy, patients with clinically overt hypothyroidism or hyperthyroidism and also Female patients with past history of oral contraceptive pills. the groups of cirrhotics with and without HCCwere followed up until the study termination (either 12 months after recruitment or PVT occurrence and were assessed at baseline and after 3 months by CBC, prothrombin time (PT), liver function analysis, and abdominal Doppler US. Final diagnosis of PVT based on Doppler US as shown in Figures 1 and 2. Computed tomography (CT), or magnetic resonance imaging (MRI) are used at the time of the enrollment for prope diagnosis of HCC. With regard to the development of PVT during follow up, we assessed, for their prognostic indication, the clinical and demographic characteristics of patients at baseline. We analyzed sex, age, platelet count, international normalized ratio, PT, plasma Annexin V level, results of abdominal Doppler US, and the MELD score between PVT and non-PVT patients.

### 2.1 Sampling and biochemistry

6 ml of blood was get from each individual (1 ml on EDTA tube for the Complete blood count, and 1.8 ml in a sodium citrate tube for measurement of plasma anxA5, PS + MP and the coagulation profile).

The citrated blood was centrifuged at 3000g for 15 min to obtain Platelet-poor plasma, and then kept at -70 °C for measurement of procoagulant activity of microparticles using a commercial enzyme immunoassay from Hyphen BioMed. Plasma anxA5 level was measured using a commercial enzyme immunoassay from Hyphen BioMed on Stat Fax 4700 Microstrip Reader l ELISA Microstrip Reader. in order to get plasma Annexin A5/PS + MP ratio. Firstly, converting plasma Annexin A5 from Mass Concentration (ng/ml) to Molar Concentration (nmol/l) by (plasma Annexin A5 concentration (ng/ml) ∗1000)/molecular weight of Annexin A5 (35.7 kDa).

### 2.2 Doppler ultrasonography screening

A color Doppler ultrasonography screening was performed by System portable ultrasonography of Toshiba SSA-320A (JUSTVISION 200). All abdominal Doppler US results were interrupted by an expert radiologist in NHTMRI.

### 2.3 Statistical analysis

All statistical analyses were analyzed SPSS (version 19; SPSS Inc., Chicago, USA) and MedCalc version 18.11. Quantitative variables are recorded as mean ± SD or median (interquartile ranges) and qualitative variables are recorded as frequencies. Comparison between the three populations (Cirrhosis without HCC group, cirrhosis with HCC group, and control group) was done through one-way analysis of variance (ANOVA). We used the Mann–Whitney U-test to analyze nonparametric data and the Student t-test for parametric data between the groups, whereas we used the χ2-test for categorical data. ROC curves were drawn to determine the capacity of independent factors in predicting risk of PVT development, AUC was obtained for each factor. A p-value lower than 0.05 was deemed statistically significant.

## 3 Results

### 3.1 Populations characteristics

91 cirrhotics patients were recruited in our study, 44 with HCC and 47 without HCC. 20 healthy individuals (control group) were recruited, too. The demographic, clinical, and biochemical characteristics of all the groups at baseline are found in Table 1.

**Table 1:** Demographic, clinical, and biochemical characteristics of enrolled patients at the baseline of the study.

Variables	Cirrhosis without HCC group (n = 47)	Cirrhosis with HCC group (n = 44)	P1 value	Control group (n = 20)	P value
Age (years)	56.8 ± 6.89	57.7 ± 5.75	0.968 (NS)	54.5 (60.5-50.5)	0.337 (NS)
Sex (M/F)	27/20	23/21	0.751 (NS)	10/10	0.587 (NS)
MELD	15.1 ± 4.57	16.1 ± 4.16	0.568 (NS)	-	-
Child–Pugh class (A/B/C)	14/18/14	12/20/12	0.733 (NS)	-	-
Portal vein thrombosis, n (%)	6 (12.7 %)	10 (22.7%)	<0.01 (HS)	0	**<0.001∗ (HS)**
ALT (U/L)	53.3 ± 15.94	51 ± 17.65	0.761 (NS)	11.9 ± 3.47	**<0.001∗ (HS)**
AST (U/L)	68.2 ± 19.51	69 ± 23.39	0.944 (NS)	15.4 ± 4.83	**<0.001∗ (HS)**
INR	1.59 ± 0.51	1.50 ± 0.47	0.542 (NS)	1.005 ± 0.49	**<0.001∗ (HS)**
PT(s)	18.62 ± 4.64	17.51 ± 4.21	0.533 (NS)	13.07 ± 0.18	**<0.001∗ (HS)**
Total bilirubin (mg/dl)	2.59 ± 2.01	2.55 ± 1.61	0.579 (NS)	0.49 ± 0.25	**<0.001∗ (HS)**
Albumin (g/dl)	2.54 ± 0.79	2.97 ± 0.72	0.098 (NS)	4.43 ± 0.59	**<0.001∗ (HS)**
Platelet count (x10^3^/cm^2^)	111.32 ± 17.63	113.04 ± 24.94	0.935 (NS)	297.7 ± 17.87	**<0.001∗ (HS)**
Portal flow velocity (cm/s)	**17.35 ± 3.10**	**16.10 ± 3.09**	**0.028 (S)**	**27.65 ± 4.25**	**<0.001∗ (HS)**
Plasma Annexin A5 (ng/ml)	**3.06 ± 0.88**	**3.89 ± 0.85**	**0.041 (S)**	**0.54 ± 0.11**	**<0.001∗ (HS)**
PS + MPs (nmol/l	**32.42 ± 7.26**	**26.7 ± 4.95**	**<0.001 (HS)**	**2.13 ± 0.76**	**<0.001∗ (HS)**
plasma Annexin A5/PS + MP ratio	**0.00415 ± 0.00105**	**0.00309 ± 0.00152**	**<0.001 (HS)**	**0.00795 ± 0.0029**	**<0.001∗ (HS)**

**Note:** p value for comparing between the three groups. p_1_ value for comparing between cirrhosis without HCC group and cirrhosis with HCC group. Values are expressed as mean ± SD, median (IQR) or n (%). ∗: statistically significant at p ≤ 0.05. The bold values are represents the statistically significant results.

### 3.2 PVT incidence in cirrhotics with and without HCC

At the endpoint of our study, PVT was detected in 16 (17.7 %) cirrhotics, 10 with HCC and 6 without HCC. the cirrhotic patients who exposed to PVT development were named as the PVT group, while cirrhotics who did not expose to PVT development were named as the non-PVT group (Table 2). cirrhotic with HCC displayed higher levels of PhosphatidylSerine bearing microparticles (PS + MPs) and plasma AnnexinA5 levels than cirrhotics without HCC and the controls. Also, cirrhotics with HCC displayed lower plasma Annexin A5/PS + MP ratio than cirrhotics without HCC and the controls (Table 1).

**Table 2:** Demographic data, clinical, biochemical, and radiological characteristics at baseline of all cirrhotic patients with and without HCC who developed PVT or not.

Variables	Cirrhosis without HCC group (n = 47)		P value	Cirrhosis with HCC group (n = 44)		P value	Total cirrhotic patients (n = 91)		P value
	PVT (n = 6)	No PVT (n = 41)		PVT (n = 10)	No PVT (n = 34)		PVT (n = 16)	No PVT (n = 75)	
Age (years)	60.0 (60.5-54.5)	59.0 (63.5-54.5)	0.633 (NS)	59.0 (64.0-52.0)	58.0 (62.0-53.0)	0.662 (NS)	59.5 (62.5-51.25)	59.00 (63-53)	0.986 (NS)
Sex (M/F)	3/3	24/17	0.656 (NS)	4/6	19/15	0.500 (NS)	7/9	35/40	0.709 (NS)
Child-Pugh class (A/B/C)	1/3/2	13/16/12	0.427 (NS)	2/4/4	10/16/8	0.806 (NS)	3/7/6	23/32/20	0.411 (NS)
MELD	18.0 ± 2.0	14.06 ± 4.90	0.136 (NS)	16.40 ± 2.70	15.13 ± 5.22	0.759 (NS)	17.0 ± 2.45	14.56 ± 5.0	0.215 (NS)
ALT	49.00 ± 16.46	53.05 ± 17.02	0.707 (NS)	52.20 ± 13.59	50.26 ± 20.18	0.854 (NS)	51.00 ± 13.62	51.75 ± 18.31	0.914 (NS)
AST	60.66 ± 21.36	69.23 ± 19.42	0.452 (NS)	67.60 ± 24.26	70.33 ± 25.24	0.835 (NS)	65.62 ± 21.90	70.50 ± 22.52	0.538 (NS)
PT	18.30 ± 2.81	18.41 ± 5.18	0.672 (NS)	16.08 ± 3.20	18.05 ± 4.73	0.570 (NS)	16.91 ± 3.07	18.24 ± 4.90	0.352 (NS)
INR	1.58 ± 0.24	1.51 ± 0.53	0.525 (NS)	1.09 ± 0.58	1.54 ± 0.49	0.173 (NS)	1.28 ± 0.53	1.53 ± 0.51	0.370 (NS)
Albumin	2.64 ± 0.77	2.00 ± 0.80	0.511 (NS)	2.98 ± 0.64	2.96 ± 0.75	0.566 (NS)	2.61 ± 0.82	2.79 ± 0.77	0.660 (NS)
Total bilirubin	4.26 ± 2.01	2.27 ± 1.91	0.125 (NS)	2.67 ± 1.81	2.18 ± 0.68	0896 (NS)	2.96 ± 1.6	2.46 ± 1.84	0.579 (NS)
Platelet count (x10^3^/cm^2^)	113.5 ± 9.35	110.73 ± 18.46	0.722 (NS)	112.1 ± 24.1	113.00 ± 25.54	0.893 (NS)	112.6 ± 19.45	111.90 ± 21.83	0.903 (NS)
Portal flow velocity (cm/s)	**14.16 ± 2.31**	**17.75 ± 2.56**	**<0.001∗ (HS)**	**13.6 ± 1.71**	**16.7 ± 2.61**	**<0.001∗ (HS)**	**13.81 ± 1.91**	**17.28 ± 2.62**	**<0.001∗ (HS)**
Plasma annexin A5 (ng/ml)	**3.30 ± 0.57**	**3.02 ± 0.91**	**0.439 (NS)**	**3.35 ± 0.65**	**3.52 ± 0.91**	**0.574 (NS)**	**3.22 ± 0.62**	**2.91 ± 1.1**	**0.06 (NS)**
PS + MP (nmol/l)	**36.6 ± 1.02**	**25.35 ± 3.39**	**<0.001∗ (HS)**	**40.01 ± 0.85**	**30.81 ± 6.78**	**<0.001∗ (HS)**	**38.73 ± 1.92**	**22.19 ± 10.58**	**<0.001∗ (HS)**
plasma Annexin A5/PS + MP ratio	**0.00235 ± 0.0005**	**0.00374 ± 0.0009**	**<0.001∗ (HS)**	**0.00239 ± 0.00047 2.45**	**0.00342 ± 0.00079**	**<0.001∗ (HS)**	**0.00238 ± 0.00047**	**0.00451 ± 0.0023**	**<0.001∗ (HS)**

**Note:** Values are expressed as mean ± SD, median (IQR) or n (%). The bold values are represents the four tested factors in ROC Curve analysis.

### 3.3 Clinical differences between the two groups

No statistically significant differences were found between PVT group and Non- PVT group in all cirrhotics, cirrhotics without HCC and cirrhotics with HCC at baseline regarding sex, age, albumin, ALKB, ALT, AST, GGT, T-Bilirubin, PT, INR, Platelet count and Plasma annexin A5 (all P > 0.05) (Table 2). Statistically significant differences were found between PVT group and Non- PVT group in all cirrhotics, cirrhotics without HCC and cirrhotics with HCC at baseline regarding plasma Annexin A5/PS + MP ratio, PS + MPs, and Portal flow velocity (all P < 0.05) (Table 2).

### 3.4 Plasma annexin A5/MP ratio had the largest AUC in predicting PVT development in total cirrhotics with and without HCC

Our findings revealed that Plasma Annexin A5/MP ratio was significantly related to development of PVT in cirrhotics with and without HCC (Table 3).

**Table 3:** Validity Plasma Annexin A5/MP ratio, PS + MPs level, Portal flow velocity, Plasma Annexin A5 level in prediction of portal vein thrombosis in cirrhotic patients without HCC; ROC curve analysis.

	Cirrhosis without HCC group			
	Plasma Annexin A5/MP ratio	PS + MPs level	Portal flow velocity	Plasma Annexin A5 level
	≤0.002808933	>35.3 (nmol/l)	≤15 (x10^3^/cm^2^)	≤3.1 (ng/ml)
Sens.	100%	83.33%	66.67%	50%
Spec.	92.68%	97.56%	78.05%	53.66%
+PV	66.7%	80%	30.8%	13.6%
-PV	100%	95.2%	94.1%	88%
AUC	0.951 (0.846-0.993)	0.917 (0.840-0.991)	0.854 (0.720-0.940)	0.571 (0.419-0.714)

**Table 4:** Validity Plasma Annexin A5/MP ratio, PS + MPs level, Portal flow velocity, Plasma Annexin A5 level in prediction of portal vein thrombosis in cirrhotic patients with HCC.

	Cirrhosis with HCC group			
	Plasma Annexin A5/MP ratio	PS + MPs level	Portal flow velocity	Plasma Annexin A5 level
Cut-off	≤00277264	>38.7 (nmol/l)	≤15 (cm/s)	>3.2 (ng/ml)
Sens.	100%	90%	90%	80%
Spec.	76.47%	85.29%	61.76%	55.8%
+PV	55.6%	64.3%	40.9%	43.8%
-PV	100%	96.7%	95.5%	93.5%
AUC	0.921 (0.821-0.920)	0.854 (0.715-0.942)	0.828 (0.684-0.925)	0.566 (0.408-0.715)

**Table 5:** Validity Plasma Annexin A5/MP ratio, PS + MPs level, Portal flow velocity, Plasma Annexin A5 level in all cirrhotic patients.

	Total cirrhotic patients			
	Plasma annexin A5/MP ratio	PS + MPs level	Portal flow velocity	Plasma Annexin A5 level
Cut-off	≤0.002808933	>35.6 (nmol/l)	≤15 (cm/s)	>3.2 (ng/ml)
Sens.	100%	87.5 %	81.25 %	62.5%
Spec.	87.37 %	85.26%	70.67%	67.37%
+PV	57.1%	50 %	37.1%	24.4.3%
-PV	100.0%	96.5 %	94.6%	91.4%
AUC 95% CI	0.919 (0.808-.947)	0.876 (0.790-0.936)	0.842 (0.751-0.910)	0.509 (0.402-0.615)

**Figure 3:**
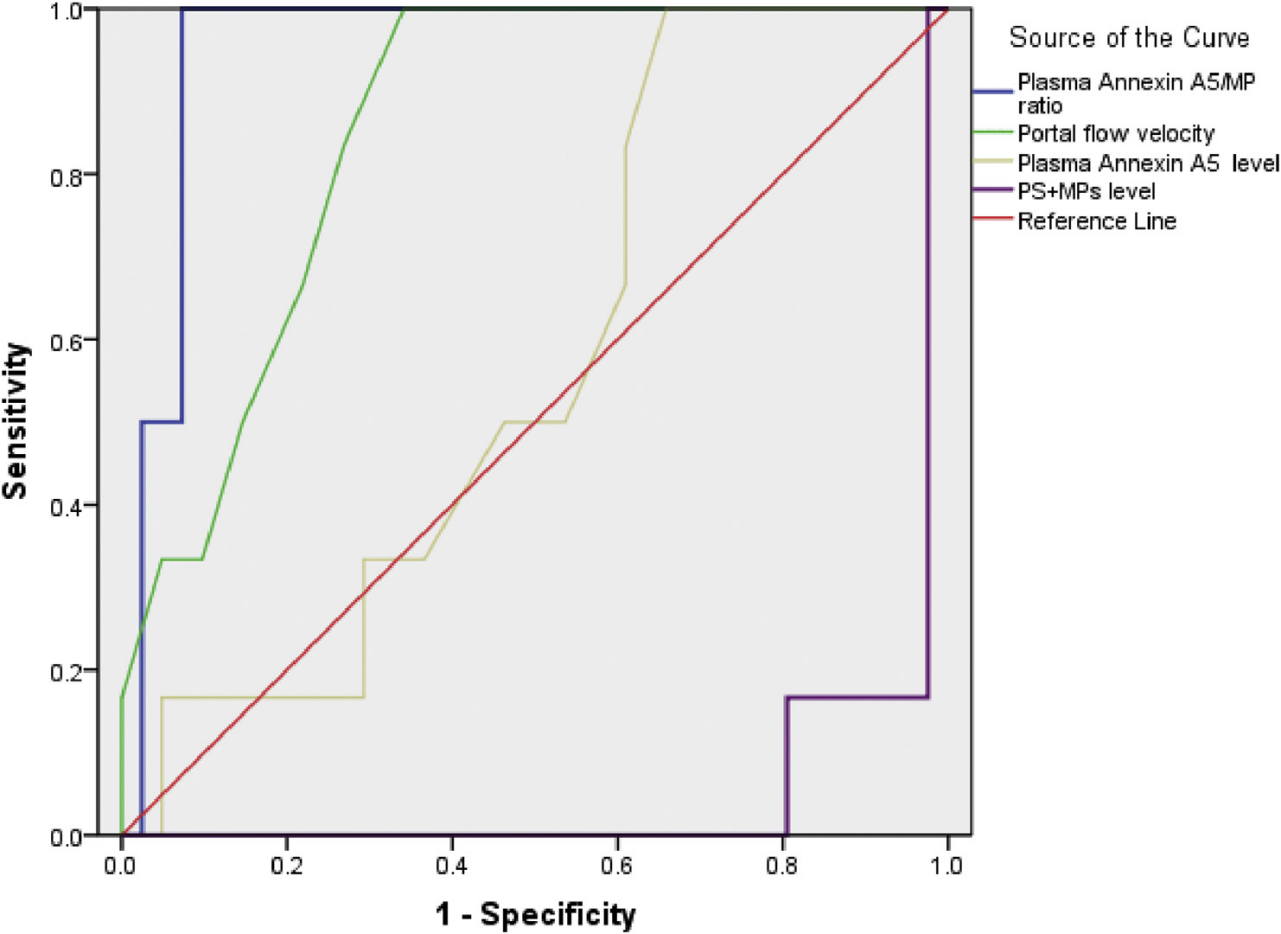
ROC curve for annexin A5/MP ratio, PS + MPs level,Portal flow velocity and Plasma Annexin A5 level in Cirrhosis without HCC group.

Using the ROC curve in Cirrhosis without HCC group, annexin A5/MP ratio had the largest AUC, 0.951, followed by PS + MPs level, 0.917, Portal flow velocity, 0.854, Plasma Annexin A5 level, 0.571 (Table 3, Figure 3).

**Figure 4:**
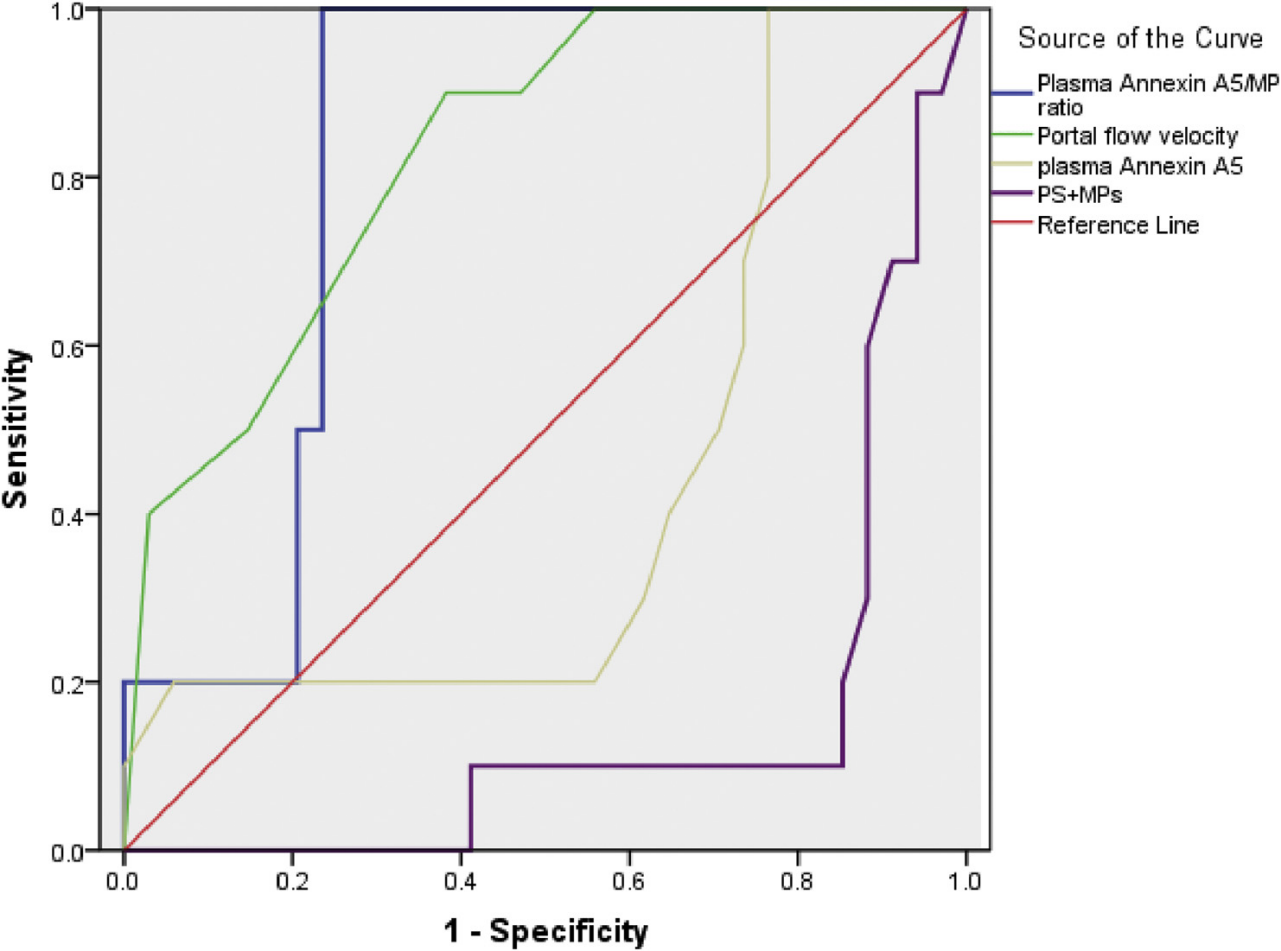
ROC curve for annexin A5/MP ratio, PS + MPs level,Portal flow velocity and Plasma Annexin A5 level in Cirrhosis with HCC group.

**Figure 5:**
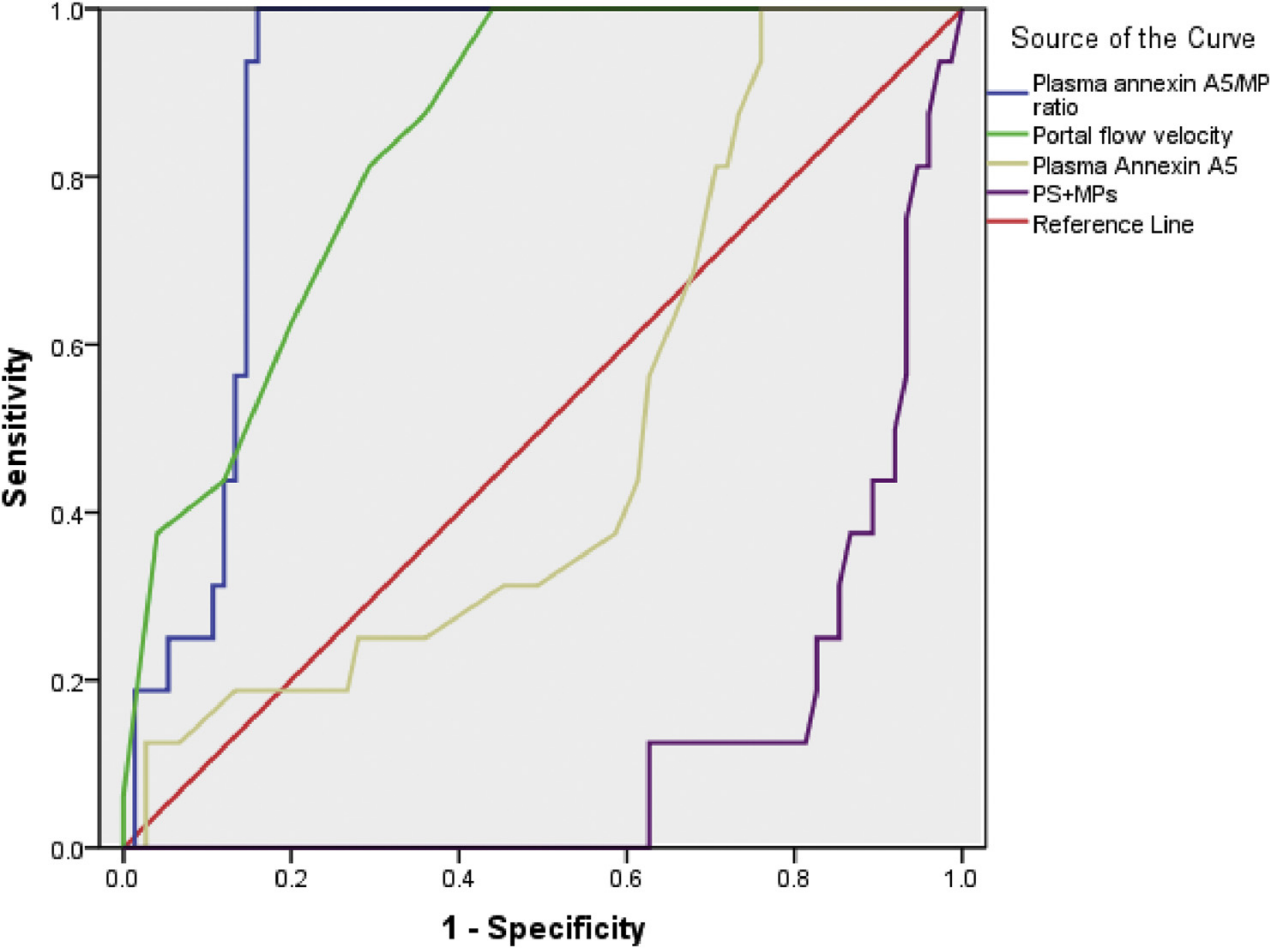
ROC curve for annexin A5/MP ratio, PS + MPs level,Portal flow velocity and Plasma Annexin A5 level in Total cirrhotic patients.

Using the ROC curve in Cirrhosis with HCC group, annexin A5/MP ratio had the largest AUC, 0.921, followed by PS + MPs level, 0.854, Portal flow velocity, 0.828, Plasma Annexin A5 level, 0.566 (Table 4, Figure 4).

Using the ROC curve in Total cirrhotic patients, annexin A5/MP ratio had the largest AUC, 0.919, followed by PS + MPs level, 0.876, Portal flow velocity, 0.842, Plasma Annexin A5 level, 0.509 (Table 5, Figure 5).

## 4 Discussion

In this study,1 2-months incidence of PVT was 17.7 % in total cirrhotics (12.7 % in cirrhotics without HCC and 22.7% in cirrhotics with HCC). This conclusion is in line with Abdel-Razik et al [21], Zanetto et al. [8] and Zocco et al. [22] who reported that PVT incidence in cirrhotics range from 16 % to 24%. The tumor cells can activate the coagulation system which in turn leads to prothrombotic and hypercoagulable state of malignancy so there is a higher incidence of PVT through cirrhotics with HCC [23]. In this study, we showed that cirrhotics with HCC had increased PS + MPs, plasma Annexin A5 levels and lower plasma Annexin A5/PS + MP ratio compared to cirrhosits without HCC and healthy controls. Additionally, cirrhotics without HCC had significantly higher levels of PS + MPs, plasma Annexin A5 and lower plasma Annexin A5/PS + MP ratio compared to healthy controls. In support of this result, ANXA5 shows tumor promoter activity in the majority of many kinds of tumor, including HCC [24]. Moreover, Zhuang and Sun *et al* demonstrated that ANXA5 expression was elevated in HCC compared with normal liver tissues [25, 26]. Our findings match with previous results exhibiting that Hepatitis C patients with HCC had higher microparticles levels compared to Hepatitis C patients without HCC a and these microparticles levels change dynamically after surgery [27]. We confirmed the presence of microparticles in cirrhotic liver compared to healthy controls, likely resulting from systemic inflammation and liver cell damage [28], so asserting microparticles role in mediating pro-inflammatory reactions, endothelial injury, and coagulation activation that are included in the liver fibrosis process. Moreover, cirrhotics with HCC who developed PVT showed significantly higher levels of PS + MPs than cirrhotics who did not. No difference in levels of annexin A5 was found between cirrhotics who developed PVT and cirrhotics who did not. This result is in aggreement with Van Heerde et al [29] who concluded that the increased plasma annexin A5 level in SLE patients isn't an indicator of vascular damage. Increased annexin A5 levels might reflect an increased apoptosis rate in these patients. cirrhotics with and without HCC who developed PVT showed significantly lower plasma annexin A5/MP ratio than cirrhotics with and without HCC who did not. In our hypothesis, As phosphatidyl exposure increase due to increased level of circulating microparticles in cirrhotics with and without HCC, anenxin-A5 may be secreted by platelets and endothelial cells into the circulation as a physiological response to inactivate the elevated levels of PS bearing MPs produced in these patients but the increase in anenxin-A5 level isn't equivalent to the increase in PS bearing MPs levels. The equilibrium between plasma annexin A5 and PS bearing MPs levels is defected.

Until now, portal flow velocity predictive value in cirrhotics concerning PVT still heatedly debated. In this work, reduced portal flow rate was associated with the PVT group in comparison to the non-PVT group, which recommended that reduced portal flow rate might be an independent risk factor for the development of PVT. This conclusion is in agreement with the conclusion of Stine et al. who detected that portal flow rate >15 cm/s is highly critical factor to predict risk of PVT progression. However, different study found that the portal flow velocities in the PVT group and Non-PVT group were not significantly different [30].

## 5 Conclusion

Risk factors of PVT development in HCV cirrhotics with and without HCC are complex. Our data suggest that disproportion between plasma Annexin A5 and PhosphatidylSerine bearing microparticles (PS + MPs) levels could be factor that lead to PVT development but these results can't be generalized and are not sufficient to confirm PVT development due to the small sample size. So, more investigations are needed to confirm these results.

## Declarations

### Author contribution statement

B. El sayed Eysa: Conceived and designed the experiments; Contributed reagents, materials, analysis tools or data.

M. Mohamed: Conceived and designed the experiments; Wrote the paper.

W. Serag: Performed the experiments; Contributed reagents, materials, analysis tools or data.

B. Mohamed: Performed the experiments; Analyzed and interpreted the data; Wrote the paper.

### Funding statement

This research did not receive any specific grant from funding agencies in the public, commercial, or not-for-profit sectors.

### Competing interest statement

The authors declare no conflict of interest.

### Additional information

No additional information is available for this paper.
